# Goats’ Performance in Unsolvable Tasks Is Predicted by Their Reactivity Toward Humans, but Not Social Rank

**DOI:** 10.3389/fpsyg.2020.00150

**Published:** 2020-02-07

**Authors:** Naoya Yoshida, Naoko Koda

**Affiliations:** Graduate School of Agriculture, Tokyo University of Agriculture and Technology, Fuchu, Japan

**Keywords:** communication, goat, human–animal relationship, individual characteristic, social cognition

## Abstract

In order to clarify the ability of animals to communicate with humans, it is necessary to examine the behaviors of animals directed at humans, taking into account individual differences. This study investigated whether the behaviors of goats (*Capra hircus*) can be predicted when given an unsolvable task. Two experiments were performed in a paddock using 16 domesticated goats. In Experiment 1, behavioral tests were conducted to determine the goats’ social rank and reactivity toward a stranger. In Experiment 2, the goats’ behaviors in an unsolvable task and two control conditions in which either only a human or bucket was presented were examined. The behaviors of the goats were video-recorded and compared between the conditions. Then, we examined whether the behaviors of goats in the presence of both the human and unsolvable task can be predicted from the scores for social rank and reactivity toward humans. Compared with the control conditions, the goats increased physical contact with the human, but did not increase gazing. It is possible that differences in individual characteristics and long-term experiences with humans can lead to differences in human-directed behaviors of animals. Although the social rank order of the goats was clearly linear, there was no correlation between their behaviors in the unsolvable task and their social rank. The goats that tended to interact with the stranger in Experiment 1 were more likely to approach and establish contact with the human in the unsolvable task than goats that reacted more averse toward humans. There was no association between the level of reactivity toward the stranger and the goats’ involvements in the unsolvable task. Therefore, it is possible that the goats which increased interactions with humans did not necessarily have low motivation to engage in unsolvable tasks, but relied on humans as a means of communication. In conclusion, the behavioral changes and its diversity as the responses toward short-term changes in the environment, such as the presence or absence of humans and unsolvable tasks, were related to differences in individual behavioral characteristics (i.e., reactivity toward humans).

## Introduction

In order to improve human–animal relationships and animal welfare, increasing attention is being paid to the study of social cognitive abilities of domestic animals that are related to communicative behaviors toward humans. For example, in object choice tasks, dogs can use human gestures, such as pointing or gazing without training in order to select a container with food from two containers (Miklósi et al., 1998). This ability is more advanced in dogs than human-raised wolves (Miklósi et al., 2003). This suggests that domestication has influenced this ability. Cats, experimentally domesticated foxes, and horses can also use social gestures of humans, such as pointing, in object choice tasks (cat: Miklósi et al., 2005; fox: Hare et al., 2005; horse: Maros et al., 2008; Proops et al., 2010). Thus, not only dogs, but other companion animals and livestock, have high levels of social cognitive abilities to respond to signs from humans.

So as to understand the abilities of animals to communicate with humans, it is necessary to examine not only their responses to social cues from humans, but also their communicative signals to humans. These signals include visual interactions, such as gaze alternations directed at humans and objects that animals want to access, and tactile interactions with humans (Krause et al., 2018). They contribute to attracting human attention (Marshall-Pescini et al., 2013; Alterisio et al., 2018). These behaviors can be examined using unsolvable tasks. During these tasks, we examine how animals interact with humans in the presence of food that they cannot access (Miklósi et al., 2000; Ringhofer and Yamamoto, 2016). Animal behaviors during these tasks have been studied in several species as shown below. Dogs can communicate the presence of food to humans by performing a “showing behavior,” such as gaze alternations and vocalizations (Miklósi et al., 2000). Horses can also gain the attention of humans in similar situations by performing gaze alternations (Malavasi and Huber, 2016), and they can change the way they communicate with humans depending on whether or not the humans know about the presence of food (Ringhofer and Yamamoto, 2016).

It has further been suggested that individual characteristics, particularly reactivity toward humans, may relate to the behaviors of animals directed at humans and to the problem-solving behaviors. For example, dogs with friendly relationships with humans tended to gaze at humans for a longer time when given an unsolvable task (Jakovcevic et al., 2012). In addition, both dogs and horses were similarly less likely to be involved in problem-solving tasks as their interest in humans increased. Dogs with good relationships with humans had lower problem-solving abilities (Topál et al., 1997). The problem-solving ability of horses is negatively correlated with their degree of interest in humans (Lesimple et al., 2012).

The present study focused on goats (*Capra hircus*). Goats have been domesticated since about 10,000 B.C. and have a long history of interaction with humans (Zeder and Hesse, 2000). Unlike dogs, which are companion animals, and horses, which have been used for transportation, goats have not been domesticated for the purpose of communicating with humans, but rather as food resources. However, goats have been shown to have a high level of social cognitive ability with respect to humans. For example, goats that were naïve to object-choice tasks can use pointing or touching as a signal (Kaminski et al., 2005), and goats can use the orientation of a human’s body to understand their attentional state (Nawroth and McElligott, 2017). In addition, goats, like dogs and horses, exhibit gaze alternations in accordance with the human’s attentional state during unsolvable tasks (Nawroth et al., 2016), indicating that they have high communication abilities toward humans. As far as the authors know, few studies have examined the differences in behaviors of goats during unsolvable tasks due to differences in individual characteristics. For example, Langbein et al. (2018) showed that short-term handling did not affect goats’ human-directed behaviors. However, it is predicted that the behaviors will vary for the following characteristics of goats. Goats have a clear social hierarchy, which affects their behaviors. Research has indicated that the choice of food for a low-ranking individual depends on whether it has been attacked by a high-ranking individual (Kaminski et al., 2006). Like goats, cattle have also been domesticated for food, and their coping strategies differ depending on rank, with high-ranked cattle entering a handling chute earlier than other cattle (Solano et al., 2004). Thus, we can say that the behaviors of low-ranked individuals depend on other individuals’ behaviors, while high-ranked individuals are more assertive than low-ranking individuals. In addition, it has been suggested that goats’ individual characteristics can be associated with their learning abilities; for example, goats with less sociability toward conspecifics perform better in visual discrimination tasks in which they select the correct cup of different color than with high sociability. Goats which are less exploratory perform better in non-associative cognitive tasks in which they track hidden objects than subjects with higher exploration behavior do (Nawroth et al., 2017). These findings suggest that individual characteristics may relate to goats’ behaviors not only among their own species, but also in their relationship with humans. Based on these knowledge and findings from previous research with dogs and horses (Jakovcevic et al., 2012; Lesimple et al., 2012), it is expected that individual characteristics, such as reactivity toward humans, are related to the behaviors of goats in unsolvable tasks. By comparing the behaviors of various animals during unsolvable tasks, we can determine whether they reflect differences in their relationships with humans during domestication. In addition, it can lead to the clarification of how individual characteristics are related to the flexibility of behaviors in a given environment in the process of domestication.

The purpose of this study was to investigate the behaviors of goats in an unsolvable task and determine whether their behaviors could be predicted from individual characteristics. In Experiment 1, goats’ social rank order and reactivity toward a stranger were examined. In Experiment 2, the goats’ behaviors in an unsolvable task and two control conditions in which either only human or bucket was presented (human-only condition, food-only condition, and human + food condition) were examined. Then, we compared the goats’ behaviors in these conditions and investigated whether their behaviors in the presence of the human and unsolvable task could be predicted from individual characteristics. We expected the goats to increase their engagement with the human and decrease their engagement with the bucket when both the human and food bucket were present, compared to when only the human was present, as well as horses (Ringhofer and Yamamoto, 2016). In addition, previous studies suggest that individuals with high rankings are likely to be more active in the task than with low ranking (Solano et al., 2004; Kaminski et al., 2006). We predicted that if this characteristic was reflected in individual observations, high-ranked individuals will be more actively involved in the unsolvable task than low-ranked individuals in the presence of the human and unsolvable task. Furthermore, we predicted that the more goats actively interact with the stranger, the more likely they are to rely on the human and the less likely they are to attempt the unsolvable task.

## Materials and Methods

### Subjects and Housing

The experiments were conducted in the goat house at Tokyo University of Agriculture and Technology. Seventeen adult goats (15 females and 2 castrated males, 1–10 years old) were used. The goats were Japanese native breeds (*Shiba yagi* and *Tokara yagi*), which are easy to handle because of their small size (20–35 kg) and tameness. The goats lived in two groups of 7 and 10 animals. Students at the university fed and cleaned the house twice a day, in the morning and evening. All goats had no experience in training or participating in research other than for veterinary purposes, but all were used to human presence.

**FIGURE 1:**
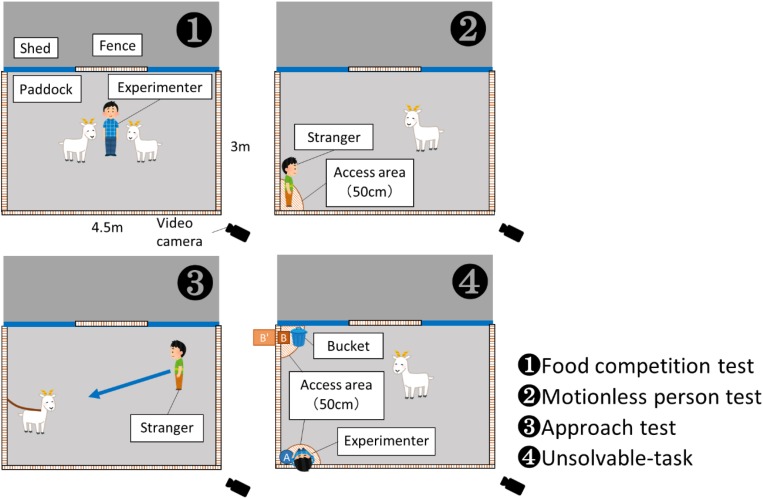
Sketches of scenarios for Experiments 1 (panels ❶–❸) and 2 (panel ❹). The experiments were conducted in the same paddock (3 m × 4.5 m). The access area (50 cm) was established to record the duration of proximity by goats. In Experiment 2, the food bucket or experimenter or both were present in different conditions.

The experiment was carried out in the paddock of the goat house (3.0 m × 4.5 m; see Figure 1), which the goats were familiar with. Except for the food competition test, the goats participated in the experiments one by one. Goats not participating in the experimental session were kept indoor in the goat house. One goat exhibited “separation anxiety behavior” [abnormal increase in phonation in the direction of conspecifics, based on the definition from Lyons et al. (1993)] and the experiment was stopped; thus data from 16 animals were used for the final analysis. A human unfamiliar with the goats, who had no experience with the goats and did not know the purpose of the study, participated as the stranger in the tests of the goats’ reactivity toward a human.

### Procedure

The experimental scenarios are provided in Figure 1. In Experiment 1, the individual characteristics of the goats (social rank and reactivity toward a human) were tested. In Experiment 2, the behaviors of the goats when they faced an unsolvable-task were examined. The experiments were conducted from May to August 2018.

#### Experiment 1 (Individual Characteristic Test)

We conducted one test to determine the social rank of the goats and two tests to determine their reactivity toward a human. The social rank test and the tests for the reactivity toward a human were conducted in random order on different days on the basis that the individual characteristics were consistent.

##### Food competition test (social rank)

The procedure to examine the social rank of the goats was based on Kaminski et al. (2006). A pair of goats was led into the same compartment, and the experimenter stood between the two animals with a piece of food (a hay cube, which was daily food). When the two goats approached the experimenter and tried to smell the food, he placed it on the ground and allowed them to access it. The procedure was repeated three times. During the intervals between these trials, the animals were allowed more than 30 s to complete their swallowing and to settle down. All pair combinations in each group were tested. The test was conducted between 5 a.m. and 8 a.m., before the morning feeding, when the animals were considered to be the most willing to feed.

##### Reactivity toward human

In order to examine the reactivity toward a human, a motionless person test and an approach test (Seaman et al., 2002) were conducted. To reduce stress on the subjects, the approach test was conducted within 5 min after the motionless person test. These tests were conducted between 10 a.m. and 3 p.m., between the morning and evening feedings.

###### Motionless person test

The motionless person test (Seaman et al., 2002) was carried out in order to examine voluntary involvements with a human. The trial time was 5 min. The experiment began when the goat was released to the area where the stranger stood still, and then the experimenter left. During the trial, the stranger kept his eyes on the goat without moving his face or body. A fixed video camera was used for recording.

###### Approach test

The procedure of Søndergaard and Halekoh (2003) was used to examine the responses of leashed goats to an approaching stranger. The goat was leashed on the side of the paddock by a 1 m rope, and the test began about 30 s after the goat had settled down. The stranger approached obliquely at a pace of 1 step (50 cm) per second. If the goat remained stationary within 1.5 m, the stranger slowly brought his hand close to the face of the goat. If the goat did not escape and approached his/her nose to smell the hand, the stranger tried to touch the goat’s neck.

#### Experiment 2 (Unsolvable Task)

Using Miklósi et al. (2000) and Ringhofer and Yamamoto (2016) as references, we examined the behaviors of goats toward a human and an unsolvable task. In this experiment, three conditions were tested, depending on the presence or absence of a human (the experimenter) and food: human-only, food-only, and human + food (2 min each). In the paddock, three points, A, B, and B′, were identified: Point A was the position where the human crouched down; Point B was the position where a colorless transparent bucket with a lid containing food (a hay cube) was placed; and Point B′ was a position on the opposite side of a fence (120 cm high) from Point B. The goats could see and touch the human and bucket, but they could not get the food until the experimenter opened the lid of the bucket at the end of each trial. During the trials, the experimenter turned his body toward B and crouched on A, ignoring the goats. The goats’ behaviors were recorded with a fixed video camera. Experiment 2 was conducted between 5 a.m. and 8 a.m. before the morning feeding, when the goats were considered most willing because of food motivation. The human-only condition was conducted first and the food-only condition was conducted next. This is because if food was presented first, the goats may think it was time for feeding and their responses toward the human may change. In order to give the goats time to settle down, approximately 5 min interval elapsed between the human-only condition and the food-only condition. In order to eliminate the short-term learning effects of these conditions, the human + food condition was performed a few days later.

In the human-only condition, the behaviors of each goat toward the human were examined. The goat was released into the paddock, and the experimenter stood at B′ without the bucket and looked at the center of the paddock for 5 s. Then the experimenter entered the paddock and crouched down at A, beginning the recording. After 2 min, this condition was completed, and the experimenter left the paddock.

In the food-only condition, the behaviors of each goat toward the unsolvable task were examined. The experimenter held the food bucket at B′ and showed it to the goat for 5 s, and he then placed the bucket (with the lid on) at B. The experimenter then left the paddock and hid out of sight of the goat. After 2 min, the experimenter returned to the paddock and opened the lid of the bucket to feed the goat and maintain motivation for the bucket.

In the human + food condition, the behaviors of goats toward the human and the unsolvable task were examined. The experimenter placed the bucket with food at B′, as in the food-only condition. After the experimenter crouched down at A for 2 min, he opened the lid of the bucket and fed the goat, and the trial ended.

### Behavioral Observation

#### Experiment 1 (Individual Characteristic Test)

In the food competition test, in which the social rank was examined, the frequency with which each individual obtained food was recorded for three trials (0–3 times), and goats who obtained food more than twice were regarded as dominants. Because the goats were reared in two paddocks, the ranks in each paddock were determined. In the motionless person test, which examined the reactivity toward the stationary stranger, the duration of the three behaviors shown in Table 1 were recorded (0 to 300 s). In the motionless person test, the stranger recorded gazing as the time that the goats directed their heads toward him. In the approach test, the responses of the goats to the approaching stranger were scored as shown in Table 2 (Søndergaard and Halekoh, 2003).

**TABLE 1:** Behaviors in motionless person test and Experiment 2.

Behavior	Definition
Gazing	Turning head and ears in the direction of stimulus
Proximity	Approaching within 50 cm of stimulus
Contacting	Touching or smelling stimulus at a distance of 1 to 10 cm

*Duration of gazing, proximity, and contacting to the stimulus were recorded (Motionless person test: 0 to 300 s, Experiment 2: 0 to 120 s).*

**TABLE 2:** Scores on the approach test and their definitions.

Score	Definition
1	Goat moved away from human before the human reached 1.5 m range
2	Goat stood still when human was within 1.5 m range
3	Goat sniffed human’s hand
4	Human touched goat on the neck

#### Experiment 2 (Unsolvable Task)

Duration and/or latency of the goats’ behaviors to the human and/or bucket were recorded in the three conditions, as shown in Table 3. The definitions of the behaviors were the same as those of the motionless person test (Table 1).

**TABLE 3:** Behaviors in Experiment 2.

	Condition		
	
Behavior	Human-only	Food-only	Human + food
Gazing at human	Latency, duration	N/A	Latency, duration
Proximity to human	Duration	N/A	Duration
Contacting with human	Latency, duration	N/A	Latency, duration
Gazing at bucket	N/A	Latency, duration	Latency, duration
Proximity to bucket	N/A	Duration	Duration
Contacting with bucket	N/A	Latency, duration	Latency, duration

*Behaviors to human were recorded in the human-only condition and human + food condition, and behaviors with the bucket were recorded in the food-only condition and human + food condition. N/A indicates unrecorded items because there was no object to be compared.*

#### Statistical Analysis

IBM SPSS Statistics version 19 was used for the statistical analysis.

##### Rank calculation and extraction of key parameters for reactivity toward human (Experiment 1)

Subjects were ranked by dominance according to Barroso et al. (2000) and Alvarez et al. (2003) as follows:

D⁢(Dominance)=Number⁢of⁢goats⁢displaced⁢by⁢the⁢subject/(Numberofgoatsdisplacedbythesubject+Numberofgoatsthatdisplacedthesubject)

We examined the relationship between the behavioral variables of the motionless person test (gazing, proximity and contacting) and the scores of the approach test using Spearman’s rank correlation coefficient to extract the key parameters for the reactivity toward the stranger. The alpha level was adjusted by Bonferroni correction (α = 0.0083). The primary parameter was determined to a variable or score that was significantly correlated with all other variables or scores related to the reactivity toward the stranger.

##### Comparison of behaviors between conditions in Experiment 2 and prediction by individual characteristics

Logarithmic transformation was carried out in order to ensure normality and homoscedasticity of the latency (gazing and contacting) and duration (gazing, proximity, and contacting) of the behaviors in the three conditions of Experiment 2 (human-only condition, food-only condition, human + food condition). Differences in these behavioral variables between the conditions (human-only condition vs. human + food condition, food-only condition vs. human + food condition) were then examined by multivariate analysis of variance (MANOVA) using a general linear model. We compared the human-directed behaviors (gazing, proximity, and contacting) between the human-only condition and human + food condition and the food bucket-directed behaviors (gazing, proximity, and contacting) between the food-only condition and human + food condition. The behavioral variables were set as the dependent variables, and the conditions were set as the independent variables. Also, a multivariate regression analysis was performed to examine whether the behaviors of goats in the presence of both the human and unsolvable task can be predicted from the scores for the social rank and reactivity toward the human. We used the logarithmic transformed latency and duration of the behaviors in the human + food condition as the dependent variable and the social rank score and the main scores for the reactivity toward the human as the independent variables. Stepwise method was used for the regression analysis to eliminate non-significant parameters and to select the best fit independent variables when predicting the dependent variables.

##### Coding reliability

The first observer performed all behavioral analyses. Since the judgments of the gazing were expected to be the most confusing behavior, a second independent observer who did not know the purpose of the experiment recorded the duration of the gaze behaviors toward the human and bucket (Experiment 2) for 13% of the total trials. Spearman’s rank correlations confirmed that the recorded duration was highly correlated with that of the first observer (*r* > 0.93).

## Results

### Scores of Social Rank and Reactivity Toward Human (Experiment 1)

The group structure was organized a linear hierarchy. There was no contradiction in the overall rank relationship among individuals (i.e., if *D*_1_ is higher than *D*_2_ and *D*_2_ is higher than *D*_3_, *D*_1_ is necessarily higher than *D*_3_). Table 4 provides the Spearman’s rank correlations between the three behavioral variables of the motionless person test and the scores on the approach test. The approach test score was significantly correlated with other scores for the reactivity toward the human (duration of gazing, proximity, and contacting toward a stranger in motionless person test). Thus, the score of the approach test was used as the primary parameter for the reactivity toward the human.

**TABLE 4:** Spearman’s Correlation Coefficient (and associated *p*-values) for the duration of the behaviors in the motionless person test and the scores in the approach test (*n* = 16).

	Motionless person test		
	
	Gazing	Proximity	Contacting
**Motionless person test**			
Proximity	0.355 (0.177)		
Contacting	0.527 (0.036)	*0.906 (0.000)*	
Approach test	*0.648 (0.007)*	*0.702 (0.002)*	*0.668 (0.005)*

*α = 0.0083 (Bonferroni’s correction), the significant combinations are shown in italics.*

### Comparison of Behaviors Between Conditions Concerning Unsolvable Task and Prediction by Individual Characteristics (Experiment 2)

#### Comparison of Behaviors in Three Conditions (Human-Only Condition, Food-Only Condition, and Human + Food Condition)

We compared the human-directed behaviors between the human-only condition and human + food condition and the food bucket-directed behaviors between the food-only condition and human + food condition (regarding the data before logarithmic transformation, see Supplementary Appendix 1). Significant differences were found in the following items.

Human-only⁢condition⁢vs.human+food⁢condition

**FIGURE 2:**
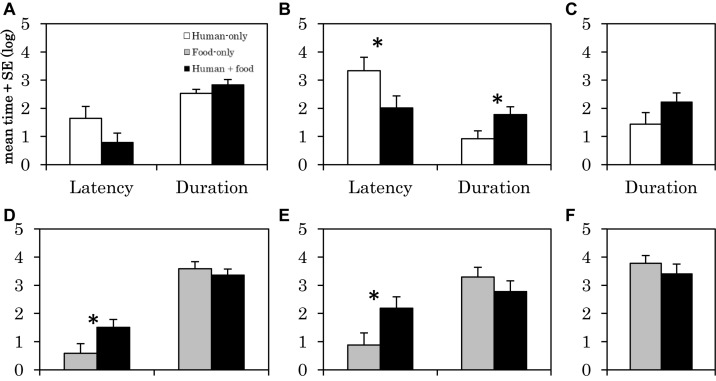
Latency and duration of the behaviors in the human-only condition, food-only condition, and human + food condition in Experiment 2. Each graph shows the time of **(A)** gazing at human, **(B)** contacting with human, **(C)** proximity with human, **(D)** gazing at bucket, **(E)** contacting with bucket, **(F)** proximity with bucket. The vertical axis of the graph represents the logarithmically transformed mean + standard error of time for each behavior. α = 0.05, ^∗^*p* < 0.05.

The latency of physical contact with the human was shorter in the human + food condition than in the human-only condition (*F*_1_,_30_ = 4.23, *p* = 0.049, Figure 2B). In addition, the duration of physical contact with the human was longer in the human + food condition than in the human-only condition (*F*_1_,_30_ = 4.623, *p* = 0.040, Figure 2B). There were no significant differences in the other behaviors (Figures 2A,C).

Food-only⁢condition⁢vs.human+food⁢condition

The latency for gazing at bucket was longer in the human + food condition than in the food-only condition (*F*_1_,_30_ = 4.328, *p* = 0.046, Figure 2D). The latency of contacting the bucket was longer in the human + food condition than in the food-only condition (*F*_1_,_30_ = 4.943, *p* = 0.034, Figure 2E). There were no significant differences in the other behaviors (Figure 2F).

#### Predictions of Behaviors in Human + Food Condition Based on Individual Characteristics

The scores of the reactivity toward the human predicted the human-directed behaviors in the human + food condition. The longer the goats interacted with the stranger in Experiment 1, the more duration they spent for proximity and contacting with the human in the presence of the human and unsolvable task (proximity: *p* = 0.004, contacting: *p* = 0.009, Table 5). The social rank scores did not predict the behaviors in the human + food condition.

**TABLE 5:** Results of multivariate regression analysis.

Dependent variables	Independent variables	*r*	*B*	*R*	*R*^2^	*F*
Proximity with human	Reactivity toward human	0.683	0.683**	0.683	0.466	12.215**
	Social rank	−0.112				
Contacting with human	Reactivity toward human	0.626	0.626**	0.626	0.392	9.018**
	Social rank	−0.177				

We used the behavioral variables in the human + food condition (latency and duration of gazing, proximity and contacting toward human and food) as the dependent variables. *r*: correlation coefficient, β: standard partial regression coefficient, *R*: multiple correlation coefficient, *R*^2^: coefficient of determination, and *F*: *F*-value. **p < 0.01.

## Discussion

This study investigated the behaviors of the goats when given the unsolvable task and whether these behaviors can be predicted from the individual characteristics of the goats. Results indicated that in the presence of both the human and bucket, the goats touched the human sooner and longer than in the human only condition. In addition, it took less time to look at and touch the bucket when only the bucket was present than when both the human and bucket were present. In other words, in the presence of the human and unsolvable task, the goats became more interested in the human and engaged more in contact with him, but were less interested in the task. We also examined whether the individual characteristics can predict the behaviors of the goats in the unsolvable task. The results showed that the goats which tended to interact with the stranger were more likely to approach and interact with the human during the unsolvable task. Conversely, it was not possible to predict the behaviors in the unsolvable task from the social rank. The results were generally in line with the expectations, except for the fact that the behaviors during the unsolvable task could not be predicted from the social rank.

Compared with the control conditions, the goats increased physical contact with the human, but did not increase gazing. The existence of gaze alternations could not be confirmed in this study because of the limitations of the condition setting. However, interestingly, when given an unsolvable task, goats performed gaze alternations to humans according to the human’s attentional state (Nawroth et al., 2016). Goats used by Nawroth et al. (2016) have experienced a lot of positive interactions with humans and circumstances in which food is inaccessible. On the other hand, the goats used in this study experienced only basic interaction with humans such as routine feeding and caring. Langbein et al. (2018) found that differences in short-term handling did not affect the behaviors of goats toward humans. However, differences in the way the goats interact with humans over a long period of time in ontogeny may have affected how the goats communicate with humans. Besides, the reactivity toward humans was associated with the goats’ behaviors during the unsolvable task. It is therefore quite possible that differences in individual characteristics, such as reactivity toward humans, and experiences with humans can lead to differences in human-directed behaviors of animals, such as gaze alternations. Previous studies showed that horses can also flexibly use the gaze alternations to convey their intentions to humans (Malavasi and Huber, 2016). Horses identify humans’ intentions and expectations by looking at the human’s visual attention status (Sankey et al., 2011). Thus, visual engagement is important not only within the species, but also in communication with humans. It is difficult to determine the essential meaning of behaviors from animals to humans, such as whether animals can acquire a human perspective and if theory of mind can be applied to animal behaviors. However, it is necessary to continue examining the degree of sophistication of human-directed signs of animals through intraspecific and interspecific comparisons.

The goats that tended to interact with the stranger were more likely to approach and contact with the human during the unsolvable task. In a previous study, dogs involved with humans for a longer time increased gazing at humans in an unsolvable task (Jakovcevic et al., 2012). Although there were differences in that the dogs used visual involvement and the goats used tactile involvements, the results indicate that the more animals react actively to humans, the more likely they were to engage with humans during unsolvable tasks. In previous studies using problem-solving situations, dogs with good relationships with humans had lower problem-solving abilities (Topál et al., 1997). In addition, the longer horses looked at a human in these situations, the longer it took to open a box with food (Lesimple et al., 2012). The unsolvable task used in our study was not comparable to results of these earlier studies using problem-solving situations, but they were consistent in that there were relationships between intensity of animals’ interest in humans and the behaviors of them when they face difficulties in the presence of humans. However, in our study, the relationship between the levels of the reactivity toward the human and the behaviors toward the unsolvable task was not confirmed. Therefore, it is possible that goats which increased interactions with humans do not necessarily have low motivations to engage in unsolvable tasks, but rely on humans as a means of communication.

The behaviors in the unsolvable task could not be predicted from the social rank. This may be due to the fact that the social rank was not reflected in the context of individual behaviors or human interactions. In particular, a review by Hartmann et al. (2017) suggests that social relationships among horses do not affect human-horse relationships. However, in the daily feeding of goats, differences in the social rank are related to aggressiveness toward humans (Aschwanden et al., 2008; Miranda-de la Lama et al., 2013). Also, dominant cattle are willing to address the task of entering the handling chute, while subordinates adopt a passive strategy (Solano et al., 2004). During handling, subordinate goats approach closer to a handler than dominant goats (Miranda-de la Lama et al., 2013). This was the first study to examine the relationship between goats’ social rank and behaviors during an unsolvable task. Based on previous studies, the way goats interact with humans may differ according to the social rank, and thus the way they attempt certain cognitive tasks may differ according to the social rank. Future studies should examine the relationship between the social rank and animal cognitive ability by examining how animals cope with cognitive tasks while in groups, as well as focusing on individual behaviors.

When the overall results of this study are interpreted in conjunction with previous studies and West-Eberhard (2005), the behavioral plasticity related to short-term changes in the environment, such as the presence or absence of humans and unsolvable tasks, was to some extent related to differences in the animals’ experiences with humans. Conversely, all domestic animals have experienced human interaction in ontogeny and have repeated their learning over generations. Part of this learning came to be incorporated into genetic information in long-term relationships with humans in domestication, and it can be considered that it was strongly reflected in the differences in behaviors in daily feeding. In particular, in the case of the acquisition of food by domestic animals, optimal strategies have been adopted according to the levels of reactivity toward humans. For these reasons, some individual characteristics may be preferentially related to behavioral plasticity and its diversity in difficult situations related to food acquisition, even if species are different (Stamps and Biro, 2016). These individual differences in the behavioral plasticity and their factors need to be examined in the future. In order to clarify them, it is necessary to investigate other characteristics such as the exploration level toward the environment and some species-specific traits, with focusing on their interaction and the behavioral syndrome (Sih et al., 2004). In addition, only a small number of animals, mostly females, were used in this study. To generalize the results, we need to study more individuals, including differences between males and females. Furthermore, although the relationship between the diversity of animal cognitive abilities and behaviors has been investigated, the direction of the relationship varies. It is therefore necessary to further explore the factors that influence the direction of the association between cognition and individual characteristics (Dougherty and Guillette, 2018). It is possible to communicate effectively with animals by providing the knowledge obtained through these studies to daily relationships with animals. It will improve the welfare of animals and humans who interact with them.

## Data Availability Statement

The raw data supporting the conclusions of this article will be made available by the authors, without undue reservation, to any qualified researcher.

## Ethics Statement

The procedure was in accordance with the Code of Ethics and Conduct of the Japanese Psychological Association. Since this study was a non-invasive experiment, it was not subject to review by the university’s ethical committee for animal experiments.

## Author Contributions

NY designed the study, performed the experiments and data analysis, and drafted the manuscript. NK proposed the idea and revised the manuscript.

## Conflict of Interest

The authors declare that the research was conducted in the absence of any commercial or financial relationships that could be construed as a potential conflict of interest.
