# Supplementary material for: Goats’ Performance in Unsolvable Tasks Is Predicted by Their Reactivity Toward Humans, but Not Social Rank
Source: Front Psychol. 2020 Feb 7;11:150. doi: 10.3389/fpsyg.2020.00150 (PMC7019027; doi:10.3389/fpsyg.2020.00150)
Supplement: Supplementary file 1 [file Table_1.docx]

Appendix 1 The mean time + standard error (seconds) of the behaviors in Experiment 2 (human-only condition, food-only condition, and human + food condition) before logarithmic transformation .

N/A indicates items that have not been analyzed because the behaviors have not been recorded.
